# Scribner’s Monthly Magazine

**Published:** 1874-04

**Authors:** 


					﻿SCRIBNER'S MONTHLY MAGAZINE
for April comes to us laden, as usual, with good things. Among the articles of
especial interest are “The Great South: A Ramble in Virginia,” illustrated;
“Christ’s Resurrection Scientifically Considered;” “Concerning Some Imperial
Booty;” “ The Health and Physical Habits of English and American Women.”
				

